# Factors influencing the induction of high affinity antibodies to *Plasmodium falciparum* merozoite antigens and how affinity changes over time

**DOI:** 10.1038/s41598-018-27361-w

**Published:** 2018-06-13

**Authors:** Muyideen K. Tijani, Sreenivasulu B. Reddy, Christine Langer, James G. Beeson, Mats Wahlgren, Roseangela I. Nwuba, Kristina E. M. Persson

**Affiliations:** 1 Cellular Parasitology Programme, Cell Biology and Genetics Unit, Department of Zoology, University of Ibadan, Ibadan, Nigeria; 2 Department of Microbiology, Tumor and Cell Biology (MTC), Karolinska Institutet, Stockholm, Sweden; 3 The Macfarlane Burnet Institute for Medical Research and Public Health, Melbourne, Victoria Australia; 4 Department of Laboratory Medicine, Lund University, Skåne University Hospital, Lund, Sweden

## Abstract

Understanding the functional characteristics of naturally acquired antibodies against *P. falciparum* merozoite antigens is crucial for determining the protective functions of antibodies. Affinity (measured as k_d_) of naturally acquired antibodies against two key targets of acquired immunity, EBA175 and PfRh2, was determined using Surface Plasmon Resonance (SPR) in a longitudinal survey in Nigeria. A majority of the participants, 79% and 67%, maintained stable antibody affinities to EBA175 and PfRh2, respectively, over time. In about 10% of the individuals, there was a reciprocal interaction with a reduction over time in antibody affinity for PfRh2 and an increase for EBA175. In general, PfRh2 elicited antibodies with higher affinity compared to EBA175. Individuals with higher exposure to malaria produced antibodies with higher affinity to both antigens. Younger individuals (5–15 years) produced comparable or higher affinity antibodies than adults (>15 years) against EBA175, but not for PfRh2. Correlation between total IgG (ELISA) and affinity varied between individuals, but PfRh2 elicited antibodies with a higher correlation in a majority of the participants. There was also a correlation between antibody inhibition of erythrocyte invasion by merozoites and PfRh2 affinity. This work gives new insights into the generation and maintenance of antibody affinity over time.

## Introduction

Despite the enormity of global financial commitment to malaria control^1,2^, malaria is still considered endemic in 91 countries with an estimated 212 million cases and 429,000 deaths in 2015^3^. Studies of the immune response in individuals living in malaria endemic areas have suggested that they acquire immunity to severe forms of malaria in early childhood while immunity to uncomplicated malaria is acquired later in childhood or adulthood, depending on the level of malaria exposure^4,5^. The critical roles played by antibodies in protective immunity against malaria was first demonstrated by the classical studies of the 1960s^6,7^ and were also confirmed later^8^. In these studies, immunoglobulins purified from malaria exposed adult blood samples or cord blood harvested from pregnant women, were used to treat both the parasitological and clinical symptoms in children suffering from malaria. Subsequently, many studies in malaria endemic regions have associated levels or breadth of antibodies to various blood-stage antigens with protection^9–12^. However, the functional attributes of protective antibodies and their protective mechanisms remain unclear.

The production of high affinity antibodies is an indication of successful priming by an antigen or vaccine and indicates that B cell clones specific to such antigens have undergone affinity maturation^13^. Studies have shown that antibody avidity (often measured as serial dilutions in inhibition ELISAs) correlates with effector functions in the elimination of bacterial infections^14,15^. Furthermore, individuals who experienced *Haemophilus influenzae* type b (Hib) vaccine failure have been shown to lack the threshold levels of antibody avidity found in individuals that were protected by the vaccine^16^. High avidity antibodies have also been shown to be responsible for protection in cases of Hepatitis B and Pneumococcal conjugate vaccines^17,18^.

In malaria, affinity of antibodies produced against merozoite antigens or antigens expressed on infected erythrocytes may play important roles in antibody-dependent effector mechanism such as erythrocyte invasion inhibition, antibody-dependent cellular inhibition (ADCI), opsonic phagocytosis, or complement fixation^19^. Indeed high avidity antibodies produced against the antigen VAR2CSA have been linked to the absence of placental malaria^20^. High affinities of antibodies against the merozoite antigens MSP2 and AMA1, quantified using surface plasmon resonance (SPR) have also been associated with protection against febrile malaria^21^. Furthermore, children with uncomplicated and asymptomatic malaria produced antibodies of higher avidity than children with complicated malaria^22^. Some studies have not found avidity of antibodies to selected merozoite antigens to be important in protection against malaria when quantified using a thiocyanate-based ELISA^23,24^. The discrepancy between protective associations found in studies could be due to differences in methods used to measure antibody affinity, or subtle differences in antigen structure. Surprisingly, a study of children vaccinated with RTS,S could not establish any correlation between antibody avidity, measured by an indirect thiocyanate ELISA elution method, and protection against clinical malaria^25^. Most of the studies examining roles of antibody affinity in protection against malaria were cross-sectional studies sampling serum antibodies from individuals that maybe at different levels of affinity maturation due to physiological or genetic differences, or even unique infection/immunological experiences. Longitudinal studies involving multiple sampling from the same individuals are required to obtain a better insight into the importance of antibody affinity and mechanisms underlying immune development in naturally exposed individuals^26^. While there are several published studies of the kinetics of antibody levels over time^27^, little has been done to examine the longitudinal kinetics of antibody affinity and factors that influence the generation of high affinity responses.

In this study we have investigated the changes in affinity over time of antibodies produced against two antigens that are targets of naturally-acquired immunity EBA175 (region RIII-V)^28^ and *Plasmodium falciparum* Reticulocyte Homologue Protein 2 (PfRh2) (construct A9)^29^. We investigated the impact of age and parasite persistence on the affinity of antibodies elicited by these two important merozoite proteins. For the first time, this study also attempted to determine the possible importance of antibody affinity in invasion inhibition using *P. falciparum* parasite strains in which the EBA175 gene has been disrupted (3D7ΔEBA175). EBAs and PfRh proteins play important cooperative roles in erythrocyte invasion^30^ and EBA175 (RIII-V) and PfRh2 have been shown to elicit invasion inhibitory antibodies in rabbits and antibodies in humans living in malaria endemic areas have been associated with protective immunity^10,29,31–33^. Due to the functional linkage between EBAs and PfRh proteins, the deletion of one or two EBA genes is usually accompanied by the upregulation of one or more PfRh proteins^32,34^. Using isolates with genetic deletion of specific EBA proteins enables the investigation of antibodies to alternative invasion pathways utilized by *P. falciparum*^19,35,36^.

Surface plasmon resonance (SPR) was recently shown to be a useful technique for estimating affinity of malaria-specific antibodies, using the dissociation constant (k_d_)^21^. SPR has been recommended for use in vaccine efficacy studies to get more accurate evaluations^37^ and it has also been shown to be suitable for use due to its efficiency in point-of-care diagnosis^38,39^. Antibody levels as determined by ELISA were also compared with SPR-measured affinity to further validate the use of SPR in measurement of affinity in malaria studies.

## Results

### Variation in affinity to merozoite antigens over time

**Figure 1:**
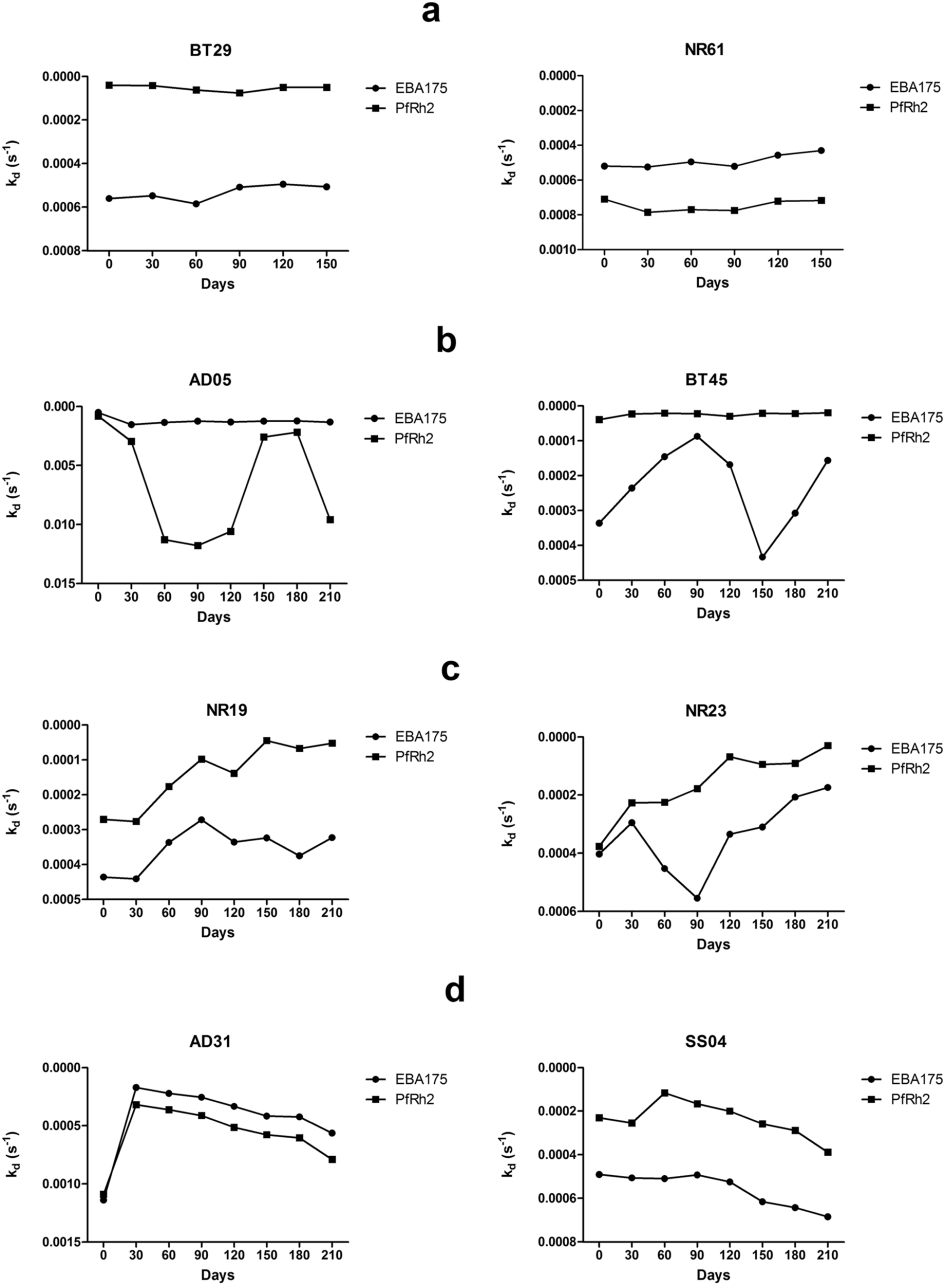
Longitudinal antibody affinity profiles of representative study participants. Affinity of antibodies to EBA175 and PfRh2 were measure by SPR. (**a**) Representative individuals in which antibody affinity to both EBA175 and PfRh2 did not change significantly throughout the study period. (**b**) Representative individuals in which antibody affinity to one of the antigens fluctuated significantly relative to affinity of day 0. (**c**) Representative individuals who exhibited the classical affinity maturation, step-wise increase in affinity with time, (note that an increase in affinity corresponds to lower k_d_ values). (**d**) Representative individuals where their antibody affinity to both proteins decrease over time. The y-axes for the graphs have been reversed.

Peripheral blood samples were collected monthly, for 6–8 months, from study participants to determine the variation over time of affinity of antibodies produced against merozoite antigens in naturally infected individuals. Affinities, expressed as k_d_ values, of antibodies specific for EBA175 and PfRh2 were measured in the plasma samples obtained from each individual. Diverse longitudinal antibody affinity profiles were observed. A majority of the participants, 79% and 67% for EBA175 and PfRh2, respectively, maintained a fairly constant antibody affinity over time (examples are shown in Fig. 1a). In some individuals, such as AD05 and BT45 (Fig. 1b), the affinity of antibodies to one of the antigens fluctuated between 50–200% of the affinity measured at day 0 (the first time point a plasma sample was collected from any individual). Other individuals, such as NR19 and NR23, showed increasing antibody affinities at least for one of the antigens, throughout the study period (Fig. 1c). There were also individuals in whom the affinity declined for most of the study period (Fig. 1d). The complete data set for all participants can be found as Supplementary Fig. S1. There was no significant association between age and the presence of antibodies with stable or changing affinity to EBA175 and PfRh2 over time.

### Differential antibody affinity to EBA175 and PfRh2

In order to explore the roles of antigen-specific factors in the induction of high affinity antibodies, we compared the antibody affinities to EBA175 and PfRh2, which have different roles in invasion pathways. Out of the 39 individuals investigated, 89% consistently produced antibodies of higher affinity to PfRh2 compared to EBA175. This difference was significant (p < 0.001) when the mean antibody k_d_ values of both proteins for all individuals was compared by Mann Whitney’s test.

### The effect of parasitemia and age on antibody affinity

**Figure 2:**
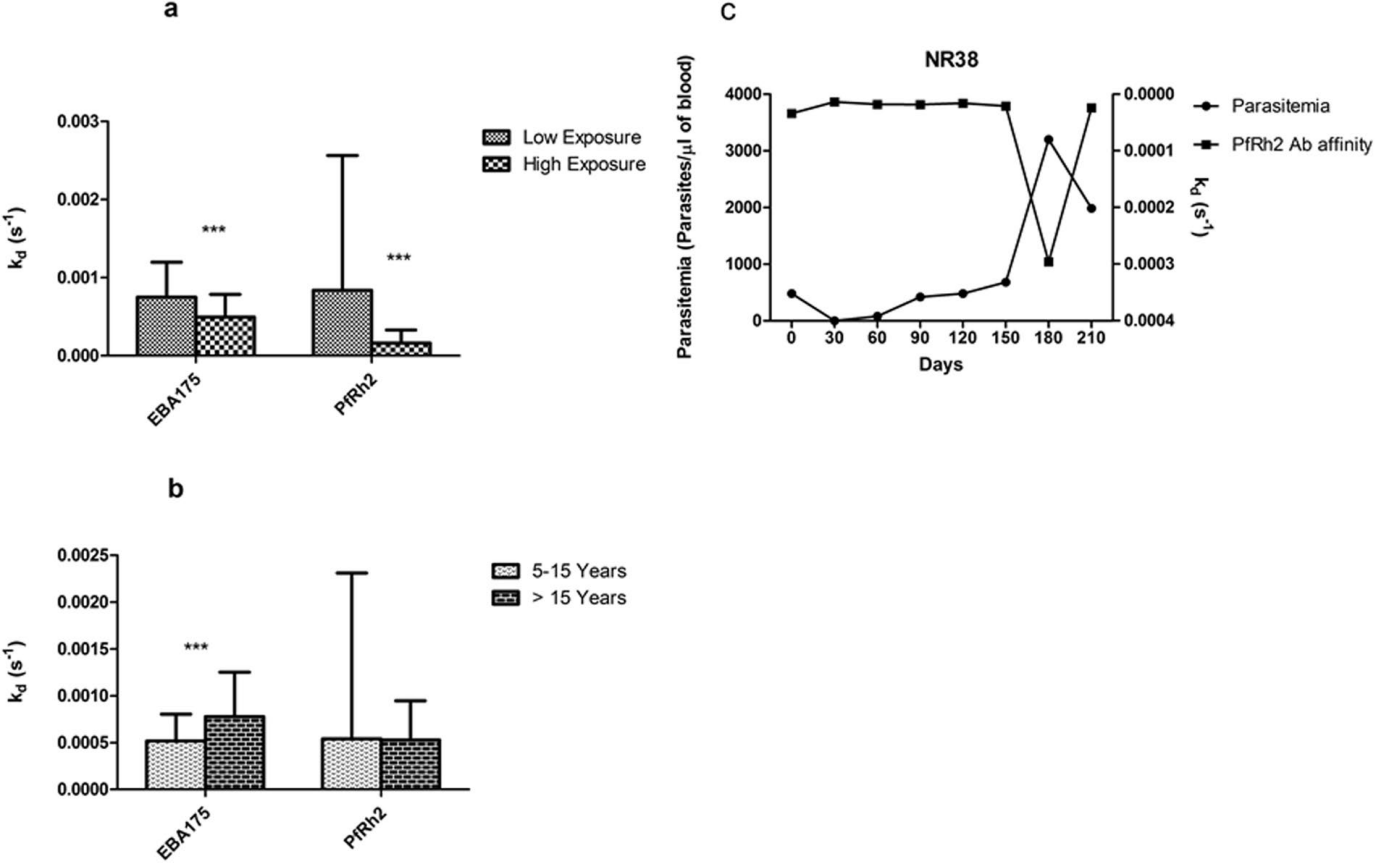
The effects of level of exposure to *P. falciparum* and age on affinity. (**a**) Comparison of antibody affinity to EBA175 and PfRh2 between individuals that encountered *P. falciparum* more frequently (median parasitemia >0 for the study period), high exposure, and individuals with low exposure. For high exposure n = 129, for low exposure n = 159; p values were calculated using Mann Whitney test (p < 0.001 = ***). Bars represent standard error of the mean. (**b**) Antibody affinity to EBA175 and PfRh2 for age groups 5–15 and >15 years. Statistical significance was determined by Mann Whitney test (p < 0.001 = ***), bar represent standard error of the mean. (**c**) Antibody affinity profile of participant NR38, 6 years old, who had the highest antibody affinity of all 288 samples on day 30. The right axis has been reversed.

Since antibody affinity is a good measure of antibody quality and may be important in immunity, it is important to identify factors associated with the generation of high affinity responses and determine if higher antibody quality is acquired with increasing frequency of parasitemia and age. The study participants were divided into two groups according to their median parasitemia for the whole sampling period. Individuals with a median parasitemia of zero were those that had fewer *P. falciparum* parasites infections, while individuals with a median parasitemia greater than zero were categorized as those with more frequent infections. The comparison of the k_d_ values in the two groups showed that individuals who were infected more frequently (high exposure) developed higher antibody affinity to both EBA175 and PfRh2 (p < 0.001 for both) (Fig. 2a). The comparison of antibody k_d_ values between the age groups showed that the younger individuals tend to produce antibodies of higher affinity to EBA175, but older individuals seem to have a slightly higher PfRh2 antibody affinity (Fig. 2b).

However, the highest antibody affinity observed throughout the study period (lowest k_d_ value, 1.39 × 10^−5^) was produced against PfRh2 by a child of age 6. It is interesting to note that at day 30 in which this child produced these high affinity antibodies was the only time point at which malaria parasites were not detected in his blood by microscopy (Fig. 2c).

### Relationship between SPR measured affinity and total IgG by ELISA

**Figure 3:**
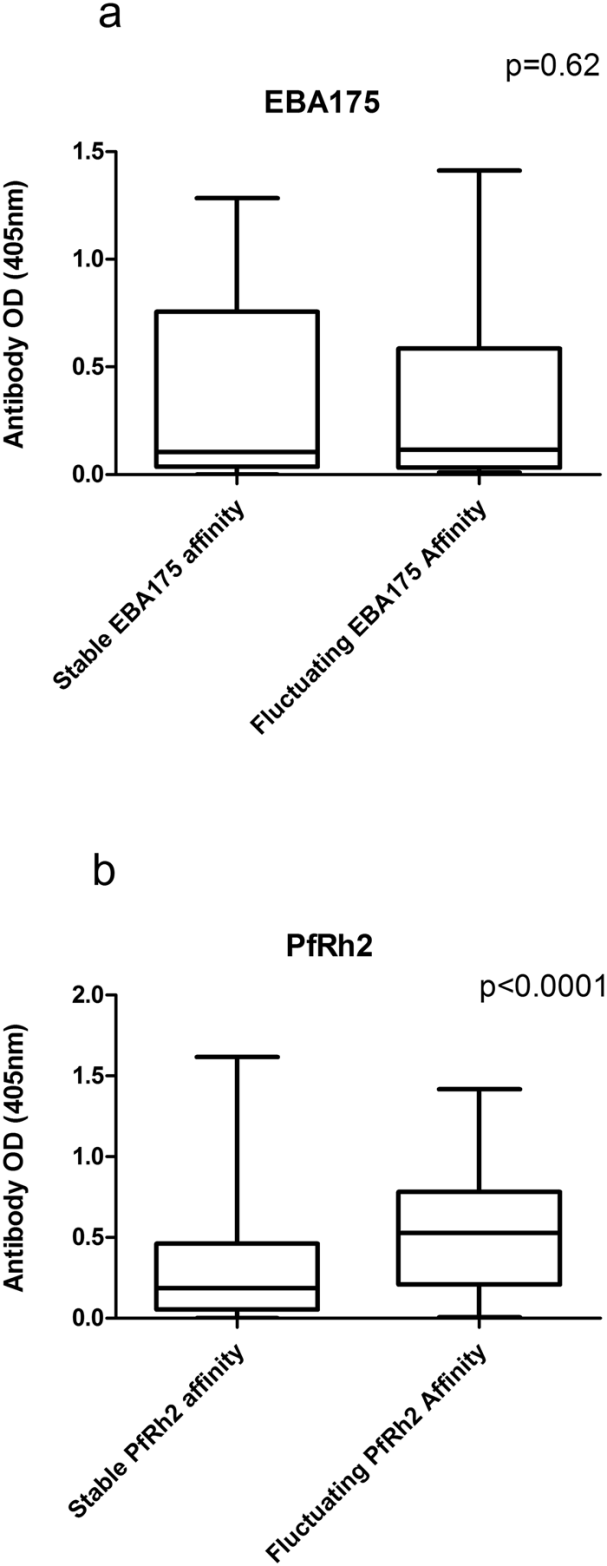
Association between antibody levels and stability of antibody affinity over time. Affinity of antibodies measured as k_d_ values were divided into two groups: stable and fluctuating. (**a**) Comparison of EBA175 antibody levels between the two groups, n = 226 for stable and n = 62 for fluctuating affinity. (**b**) Comparison of PfRh2 antibody levels between stable affinity group (n = 173) and group with fluctuating affinity (n = 115). Data were obtained from a single assay; bars represent interquartile ranges and median of samples tested in duplicate. P values were determined by Mann Whitney test.

The two recombinant antigens used for the SPR analysis in a continuous flow system were also tested in ELISA (which is a static system) to be able to compare affinity of antibodies with total IgG directed against the respective antigen. All samples from all the individuals had measurable responses for both antigens in SPR and detectable antibodies by ELISA. Generally, highly significant correlations were observed between SPR-measured affinity and total IgG (estimated by ELISA) for the PfRh2 protein from month to month throughout the study period (Table 1). However, there were no significant correlations between SPR values and ELISA-determined total IgG for EBA175 antibodies, except for one month (December) (p = 0.02). Also, there were no significant differences in EBA175 levels (OD) between individuals that had either stable or fluctuating EBA175 antibody affinities over time (Fig. 3a). However, individuals with fluctuating PfRh2 antibody affinity had significantly higher PfRh2 antibody levels than individuals with stable affinity over time (Fig. 3b).

**Table 1:** Correlation between antibody level and affinity for EBA175 and PfRh2.

Months	PfRh2. Spearman’s r, p values	EBA175. Spearman’s r, p values
July	−0.74, 0.002	−0.16, 0.48
Aug	−0.70, 0.007	0.09, 0.70
Sep	−0.80, <0.001	−0.05, 0.78
Oct	−0.78, <0.001	−0.05, 0.76
Nov	−0.80, <0.001	−0.20, 0.24
Dec	−0.81, <0.001	−0.37, 0.02
Jan	−0.76, <0.001,	−0.21, 0.19
Feb	−0.78, <0.001	−0.10, 0.54
Mar	−0.73, 0.005	−0.077, 0.8
May	−0.73, 0.007	0.03, 0.92
June	−0.75, 0.002	0.05, 0.83

**Figure 4:**
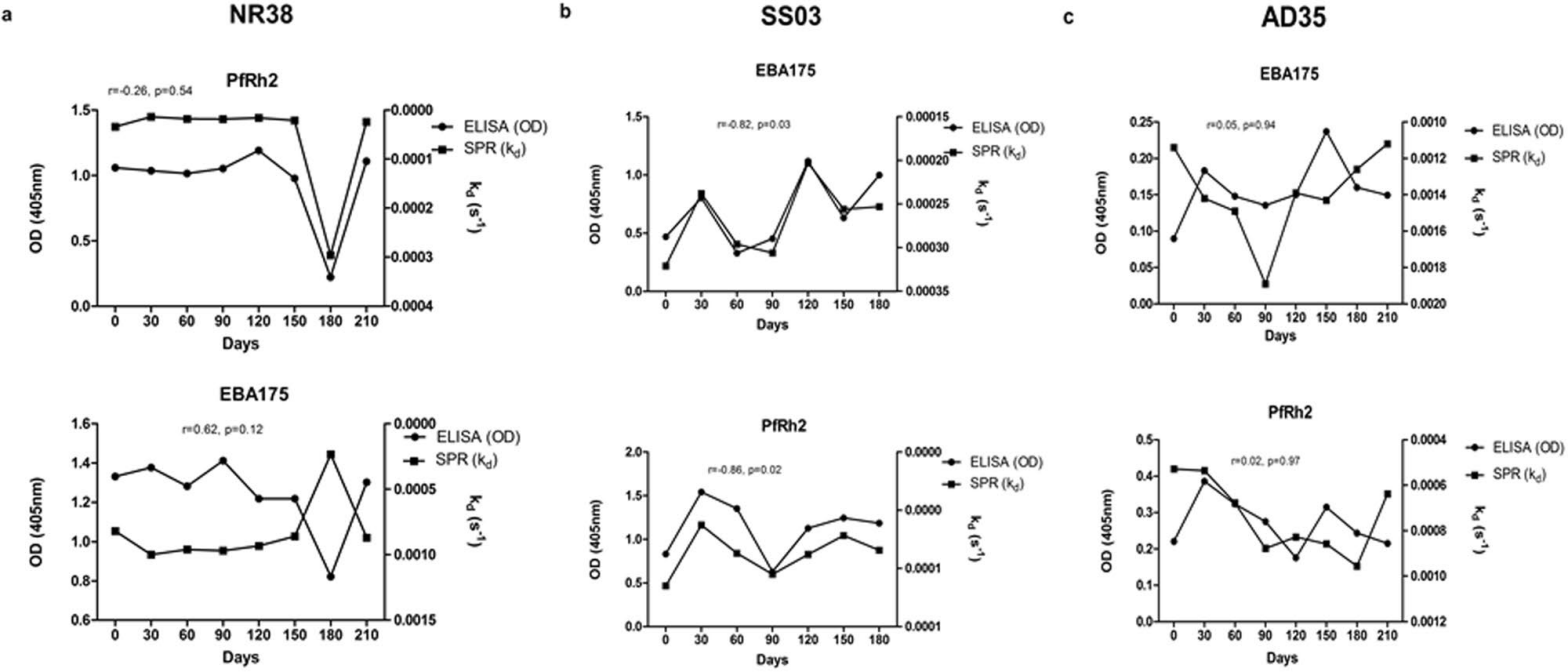
Relationship between antibody affinities (measured as k_d_ values) and ELISA-determined antibody levels for EBA175 and PfRh2. Individuals also differ in their ability to produce antibody levels with complementary affinities. (**a**) In individual NR38 there was a correlation between antibody level and affinity only for PfRh2 (the right axis has been reversed). (**b**) Participant SS03 produced antibodies levels that correlated perfectly with affinity for both EBA175 and PfRh2. (**c**) Participant AD35′s antibody levels did not correlate with affinity measured for neither EBA175 nor PfRh2. Coefficient of correlation, r, and p values were calculated using Spearman’s rank correlation test.

On an individual basis, very diverse profiles were observed: in some individuals there was a strong correlation between anti-PfRh2 antibody affinities and levels while the same individuals produced anti-EBA175 antibodies where their affinity did not correlate with levels measured by ELISA, and vice versa (Fig. 4a). There were also individuals that produced antibodies levels measured by ELISA that correlated with SPR-determined affinities to both antigens at the different time points (Fig. 4b). The last group of individuals produced anti-PfRh2 and anti-EBA175 antibodies levels that had conflicting relationship with their affinity at the different time points (Fig. 4c). These immunological differences were not affected by age (p = 0.49) or median parasitemia (p = 0.65).

### Correlation between antibody affinity and invasion inhibition

**Figure 5:**
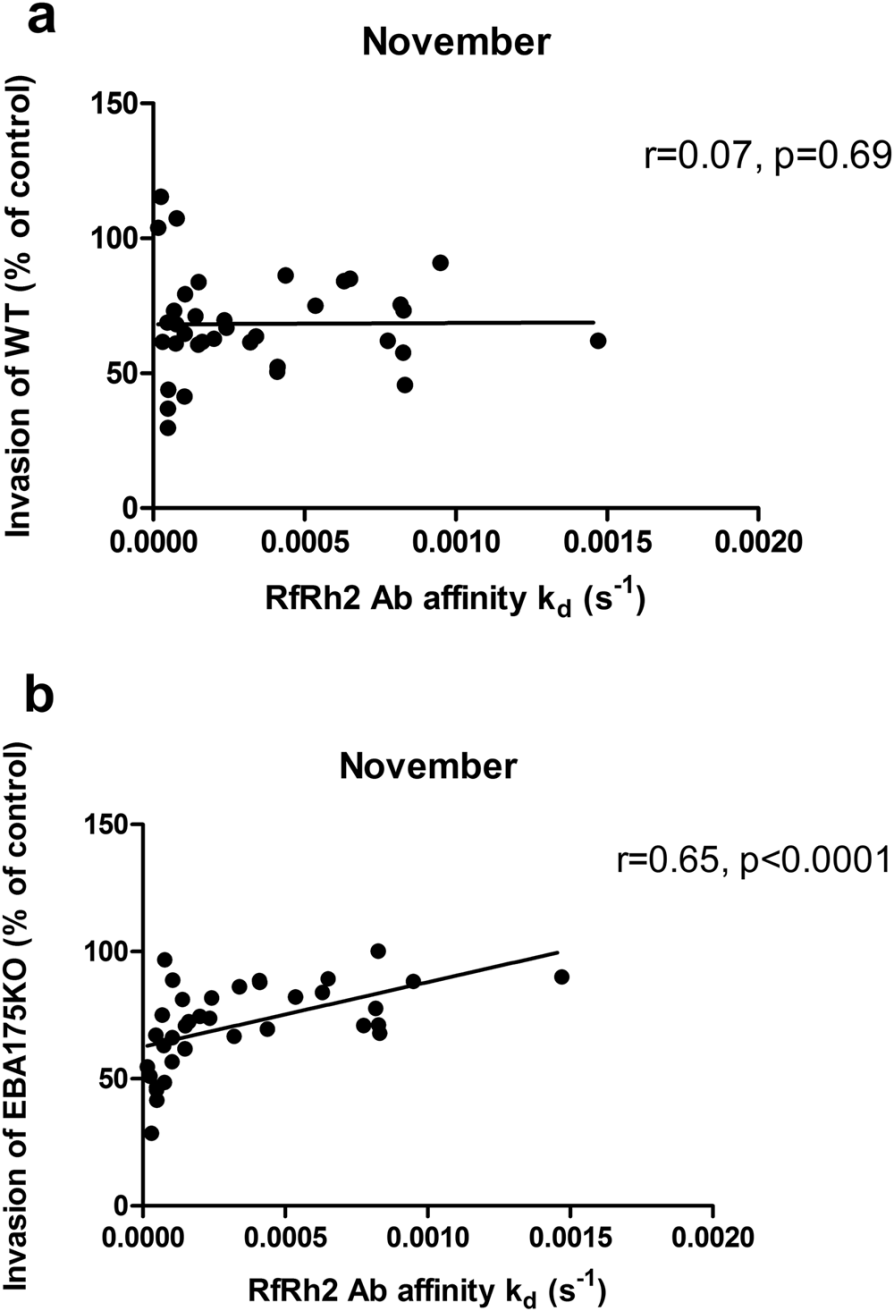
Relationship between antibody invasion inhibitory activities and affinity (measured as k_d_ values). Difference in the correlation coefficients for WT versus PfRh2 antibody k_d_ values (**a**), and 3D7ΔEBA175 versus PfRh2 antibody k_d_ values (**b**). The effect of antibody affinity to PfRh2 was more pronounced on the invasion inhibition of 3D7ΔEBA175 than of WT, possibly due to the upregulation of PfRh2 in the KO parasite. Coefficient of correlation, r, and p values were calculated using Spearman’s rank correlation test.

One of the effector functions of antibodies where affinity may play a very important role is inhibition of erythrocyte invasion by merozoites. In order to determine the possible role of antibody affinity in inhibiting the parasite invasion of erythrocytes, antibody samples were tested for inhibitory activity against *P. falciparum* parasites that had targeted disruption of EBA175 (3D7ΔEBA175) and their wild-type parental line (3D7WT). There was no correlation between percentage invasion of 3D7WT and k_d_ values of antibodies against PfRh2 for all months, but there was a correlation between percentage invasion of 3D7ΔEBA175 and PfRh2 antibodies throughout the study period (one month shown as example in Fig. 5) except for one month (February). This may indicate that upregulation of PfRh2 antigen in 3D7ΔEBA175 potentiated the effect of PfRh2 antibodies. Percentage invasion of 3D7ΔEBA175 and EBA175 antibody k_d_ values showed a significant correlation only during three of the months (March, June and November) while percentage invasion of the 3D7WT correlated with EBA175 antibody k_d_ values in two of the months (June and November).

### Reciprocal affinity modulation

**Figure 6:**
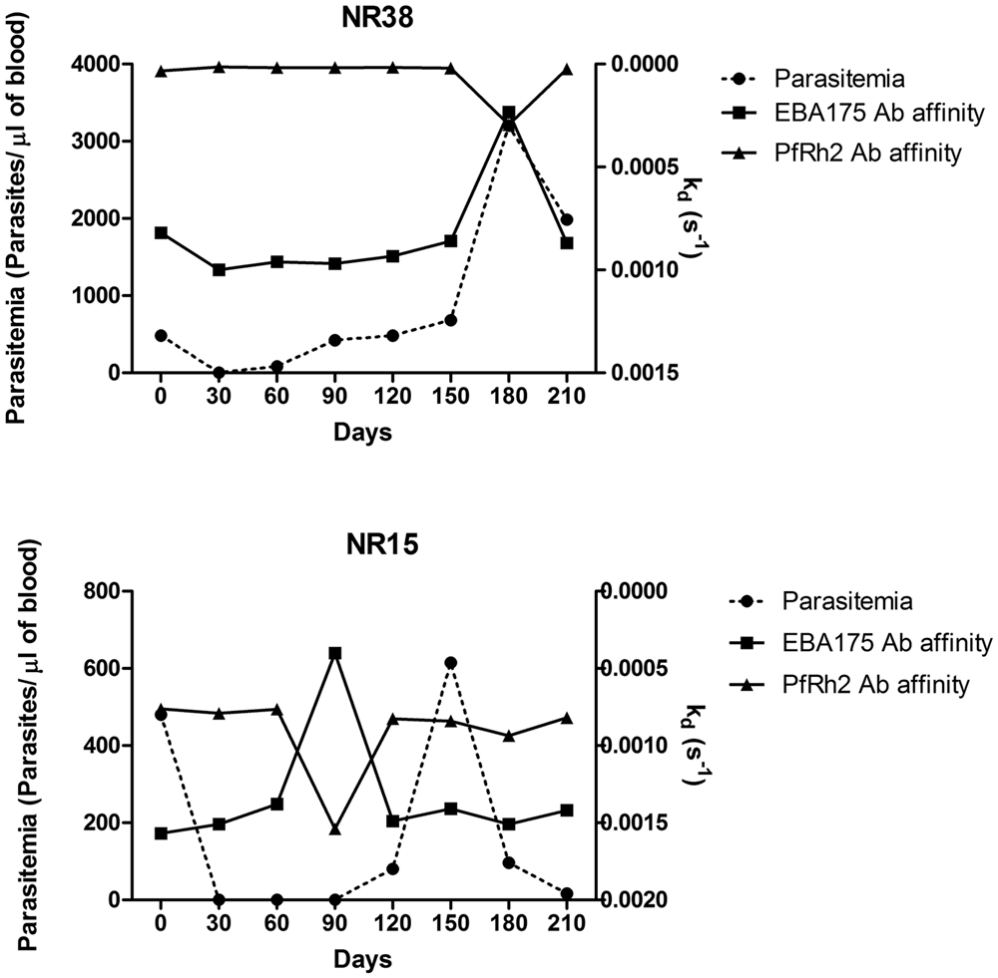
Reciprocal affinity coordination between EBA175 and PfRh2 antibody responses. Some individuals had occasions during which the fall in affinity of PfRh2 antibodies was accompanied by an increase in affinity of EBA175 antibodies: NR38 on day 180 and day 90 for NR15. Parasitemia was determined by microscopy.

The longitudinal design of this work and the study of multiple antigens enabled us to observe an event in which the reduction (at least 50% of previous month) in the affinity of antibody specific to PfRh2 was accompanied by increase (at least 50% of previous month) in the affinity of EBA175 antibodies. The k_d_ of the month in which these changes occurred, both antigens were significantly different from the mean (by One Sample t test) or the median (by Wilcoxon Signed Rank Test) of longitudinal k_d_ values for each individual that exhibited this phenomenon. This event occurred for example in individual NR38, when this individual had a peak parasitemia while the occurrence in others did not overlap with parasitemia increase (Fig. 6). This reciprocal interaction was observed in about 10% of the study participants. In all cases the affinity level returned to levels comparable to the affinity level maintained before the occurrence of this phenomenon. An important observation here is that antibody affinity (k_d_ values) and levels of anti-PfRh2 antibodies always fell together while the associated increase in affinity of EBA175 antibodies was not accompanied with increase in level of antibodies (not shown).

## Discussion

Affinity of antibodies is an important quality that has been suggested to have significant impact on the functionality of antibodies produced against various pathogens. We investigated changes in affinity of antibodies produced against two merozoite proteins that play important roles in erythrocyte invasion through a longitudinal study involving multiple sampling points of individuals living in a malaria endemic region.

Longitudinal antibody affinity profiles to EBA175 and PfRh2 varied considerably among our study participants, with some individuals exhibiting antibodies with fluctuating affinities to both or only one of the antigens, while other individuals had affinities that increased progressively through the study period. There is a scarcity of data on longitudinal profiles of antibody affinity, and the fluctuating antibody affinity observed in some of the individuals could indicate the preponderance of non-germinal center B cells (T-cell independent) that have not yet undergone classical affinity maturation. Importantly, antibody affinity to both antigens was relatively stable in a majority of the study participants, which may indicate that the antigens are largely able to stimulate the activation of B cells that have undergone affinity maturation, and have reached an antigen-specific threshold level of affinity maturation beyond which affinity cannot be increased further. This idea of antigen-specific affinity threshold seems plausible because in a majority of the individuals, affinity of PfRh2 antibodies was usually higher than that of anti-EBA175 antibodies over time. This is also in agreement with a recent study of RTS,S/AS01 vaccinees where affinity maturation of the component protein of the vaccine, circumsporozoite protein (CSP), was not observed beyond one month after the third dose^25^. A study^40^ has also demonstrated the existence of a threshold affinity level for vesicular stomatitis virus (VSC) antibodies above which no correlation was observed with protection. Furthermore, affinity maturation was not observed beyond the fourth dose of a meningococcal conjugate vaccine^41^. Apart from the differential abilities of various vaccines to elicit affinity maturation, studies of naturally-acquired antibodies to merozoite antigens have also demonstrated differences in the ability of various antigens to promote the production of high affinity antibodies. For instance, AMA1 was shown to elicit antibodies with higher affinity than MSP2^21,23^. Therefore, we speculate that the ability of PfRh2 to stimulate the production of higher affinity antibodies compared to EBA175 may be due to structural, or other, differences between these antigens. Since the expression of EBA175 and PfRh2 can vary among clinical isolates causing infections^42^, prior exposure to these antigens may also contribute to differences in affinity. We were also able to detect individuals that exhibited a pattern similar to the classical affinity maturation in which the affinity of antibodies produced against EBA175 and PfRh2 increased in a step-wise manner throughout the study period. It is possible that these individuals have not yet reached the threshold affinity level for both antigens.

The existence of an antigen-specific antibody affinity threshold may also help explain the lack of consistent relationship between age and affinity maturation of antibodies to EBA175 and PfRh2. This is in agreement with a previous study which did not find any relationship between increasing avidity of antibodies (estimated using an ELISA-elution method) to some merozoite antigens and age^24^. On the contrary, increasing avidity^22^ and affinity^21^ of malaria-specific antibodies with increasing age have also been reported, but the latter results showed a breakpoint of 16 years of age beyond which affinity remained constant. An important factor that may have occluded the impact of age on affinity maturation in this study, if there is any, is the age range of the studied participants. The youngest participant in this study was 6 years and it has been shown that somatic mutation comparable to what is usually found in the adult could be obtained in infants as early as at 8 months^43^. Therefore, the young participants in this study may have had sufficient immunological experience to be able to produce antibodies of comparable affinity to those produced by adults. This study used SPR to directly quantify antibody affinity, whereas most published studies have used an indirect ELISA elution assay using thiocyanate. Differences in methods used may be important as a prior study found no significant correlation between affinity measured by SPR and avidity estimated using the ELISA elution method^21^.

When antibody affinity was compared to levels of antibodies, there was in general better correlation for the PfRh2 antigen, which might be because of the higher affinity of antibodies to this antigen. However, individual differences could also be attributed to variability in genetic factors such as HLAs, interleukins and other immune-related genes, which are significantly associated with variation in immune responses^44^. Furthermore, environmental factors could also play a role^45,46^.

The reason for finding very few occasions of correlation between the invasion inhibition and affinity when antibodies to EBA175 were investigated might be due to the comparatively lower affinities of antibodies elicited by EBA175. This is not surprising since EBA175 antibody levels correlated poorly with functional invasion-inhibitory antibodies also in earlier studies^35,36^. There was a more consistent correlation between anti-PfRh2 antibody affinities and invasion inhibition of the EBA175 KO parasite, which might be because of up-regulation of PfRh2 in the knock-out parasite^32^. Previously, an AMA1-specific monoclonal antibody that inhibits invasion^47^ was shown to have significantly higher affinity than other AMA1-specific monoclonal antibodies that do not inhibit invasion^21^. It has also been shown that increased affinity of shark antibodies (IgNARs) induced by mutagenesis were associated with increased invasion inhibition^48^.

In some individuals we observed antibody responses to EBA175 and PfRh2 that may be reciprocally regulated. This occurrence may have been triggered by new strains of malaria parasite infections since the event often occurred after or during the peak parasitemia. The idea that infection by a new strain of *P. falciparum* could be responsible for the loss of B cells producing high affinity antibody is puzzling. However, earlier studies have suggested that heterologous infections may generate large number of plasmablasts that can lead to the displacement of established LLPCs from their niche in the bone marrow^49–51^. This is plausible, because in our study it was always the PfRh2 antibody response (with higher affinity) that was the one that diminished in all the cases of possible reciprocal regulation, suggesting that B cells specific to PfRh2 that have attained a particular level of maturation are always the target for displacement or even apoptosis. Also, both the antibody level and affinity for PfRh2 dropped simultaneously in all cases. A recent study has shown that the infection of mice with an established antibody response to influenza A virus by *P. chabaudi* could abrogate the influenza A virus antibody response temporarily by causing the apoptosis of influenza A-specific LLPCs^52^. So if the sudden drop in anti-PfRh2 level and affinity is a result of apoptosis of PfRh2-specific B cells, then the accompanying increase in affinity of EBA175 may be a form of homeostatic adjustment to maintain a steady-state of quality antibody response to malaria parasite. Further investigation of this phenomenon may yield important information that will help in understanding immune response to malaria, and the design of a multicomponent vaccine.

In conclusion, our findings provide important new insights into the acquisition and maintenance of antibody affinity to merozoite antigens, the influence of age and malaria infection, and how affinity relates to invasion-inhibitory function of antibodies. Our studies highlight the need for evaluation of antibody affinity to gain a more complete understanding of malaria immunity to inform the development of highly efficacious and long-lasting malaria vaccines.

## Materials and Methods

### Ethical issues

Written informed consent was obtained from all the parents/guardians of all children participants. Adult participants also gave consent after the details of the study had been explained to them in English or the local language (Yoruba). This study was approved by the University of Ibadan and the University College Hospital Ethical Committee (UI/IRC/06/0038) as well as the Stockholm Ethical Review Board (2013/4:8). All experiments were performed in accordance with relevant guidelines and regulations.

### Study site

This study was carried out in Igbo-Ora, South-western Nigeria. As described previously^53^, Igbo-Ora is a small town located about 100 km south of Ibadan, the capital of Oyo state. It is inhabited mainly by the Yoruba people and there are minority of Fulani herdsmen. The occupation of the people in Igbo-Ora is mainly farming, but there are also artisans and civil servants. Malaria is endemic in Igbo-Ora with a higher incidence rate during the rainy season. An entomological study in this area has demonstrated an entomological inoculation rate (EIR) of about 131 and the presence of different mosquitos’ species such as *Anopheles gambiae, An. funestus, An. arabiensis* and even an unidentified species^54^.

### Study participants and sample collection

The individuals chosen for this study were part of a longitudinal study involving 156 participants living in Igbo-Ora. During the study, 5 mL of blood samples were collected monthly from the individuals for a period that span over almost a year (July, 2009 to June, 2010) and hence cut across both the rainy and dry seasons. The plasma samples obtained were stored at −80 °C until use. At each collection time, thick and thin smears were prepared for malaria parasite detection by microscopy after staining with Giemsa. For this study, 39 individuals (equivalent to 288 samples) were selected, aged 6–57 years (median age: 16 years).

### Antigens

Recombinant merozoite antigens used in this study have been well studied^10,29^. EBA175 (RIII-V) composed of amino acid 761–1271 and PfRh2 (A9) spanning amino acid 2027–2533 (common to both PfRh2a and PfRh2b) were expressed in *E.coli* as GST fusion proteins.

### Detection of antibody levels by ELISA

ELISAs were carried out as previously described^28,35,55^. Briefly, Maxisorp microtiter plates (Nunc, Roskilde, Denmark) were coated with antigens (1 µg/ml) in 50 µl coating buffer (phosphate buffered saline, PBS, pH 7.2). Plates were incubated overnight at 4 °C, washed ×3 in PBS with 0.05% Tween 20, blocked for 2–3 hours with 200 µL/well of 10% skimmed milk in PBS/0.05% Tween 20. Plates were then washed ×3 in PBS/Tween 20 followed by addition of test plasma 1:50 in 5% skimmed milk in PBS/0.05% Tween 20 in duplicates. The plates were incubated at 37 °C for 2 hrs, washed ×3, incubated 1 hour 37 °C with 50 µl of horseradish peroxidase-conjugated goat anti-human IgG (Sigma, Germany) at 1/1000 dilution in 5% skimmed milk PBS/0.05% Tween 20. Wells were washed ×3 in PBS/0.05% Tween 20, then 50 µl of azino-bis (3-ethylbenthiazoline-6-sulphurnic acid), ABTS, solution was added. The colour was developed in the dark at room temperature for 30–40 minutes and optical density (OD) was read at 405 nm. For each plasma sample, OD from wells containing GST only was deducted from the absorbance of the EBA175 and PfRh2 GST fusion proteins. A pool of Ugandan samples and a Nigerian sample were used as positive controls; and two Swedish unexposed samples were used as negative controls on all plates to enable standardization. A plasma sample was considered positive when absorbance was higher than the mean +3 standard deviations of 12 Swedish malaria naïve samples. All longitudinal samples belonging to each individual were tested as a unit on the same plate in all cases.

### Invasion inhibition assay

The *in vitro* invasion inhibition assay is as described elsewhere^55,56^. ELISA and invasion inhibitory data have also been partly published elsewhere^55^.

### Surface Plasmon Resonance

SPR measurement of affinity was carried out as described earlier^21^. The carboxymethylated dextran surface of CM5 sensor chips (Pharmacia biosensor AB, Uppsala Sweden) were activated with a mixture of 0.05 M N-hydroxysuccinimide (NHS) and 0.05 M N-ethyl-N′-[3-diethlyaminopropyl] carbomdiimide (EDC) (Pharmacia biosensor AB, Uppsala Sweden) using an injection pulse (10 min, 5 mL/min). The EBA175 (RIII-V) and PfRh2 (A9) were immobilized by manual injection at 100 µg/mL in coating buffer (0.01 M sodium acetate buffer, pH 4.0) until the desired response units were achieved. The blocking of unoccupied activated sites on the sensor chip surface was achieved by injecting 50 µL of 1 M ethanolamine (pH 8.5) for 10 minutes. All steps were carried out in a continuous flow of HBS-EP (10 mM Hepes, 150 mM NaCl, 3 mM EDTA, 0.005% polysorbate 20) running buffer at 5 µL/min. All buffers were degassed prior to use. The binding of plasma antibodies to the immobilized antigens was performed at a constant flow rate of 30 µL/min at 25 °C. Plasma samples (always in at least two different dilutions per plasma sample, 1:7.5, and 1:15 but also at 1:30) were flowed over the bound recombinant proteins in HBS-EP buffer, pH 7.4. Usually all dilutions were included in our calculations and we always checked that the k_d_ was the same for all dilutions, to make sure that the concentration did not affect the k_d_ values. It happened for very few samples that the 1:7.5 dilution gave curves that were not smooth, probably due to thicker, fibrinous strings present in the plasma, and then only the 1:15 and 1:30 dilutions were used. GST only was used as a control that was subtracted from all samples. To prevent unspecific binding, 0.5 µg/ml of a dextran (Pharmacia biosensor AB, Uppsala Sweden) was mixed with plasma sample dilutions. The interaction of antibodies with the immobilized proteins was observed for 3 minutes of association followed by 10 minutes of dissociation. Residual antibodies that may remain attached to the immobilized antigens were removed by washing the chip with 10 mM glycine-HCl (pH 1.5) for 5 seconds at 5 µL/min before the injection of the next plasma sample. The sensor surfaces were equilibrated with the running buffer before the injection of the next sample at all time. An internal control, which was a pool of Ugandan samples, was injected after a complete set of samples (about 8 plasma samples) from each individual to test the viability of the immobilized protein. Plasma samples from individuals that exhibited abnormal binding patterns were retested to be sure that the observed binding pattern was due to the antibodies present in the samples. All samples from the same individual were tested as a unit on the same plate.

SPR analyses were carried out using Biacore T200 model (GE Healthcare Life sciences). Response was monitored as a function of time (sensogram) at 25 °C. BIAevaluation 4.1 software was used to estimate the k_d_ values.

### Statistical Analyses

Statistical analyses were performed using GraphPad Prism 5 and STATA 12.1 software. The correlations between two continuous data were performed using Spearman’s correlation test. The comparison between antibody affinity to EBA175 and PfRh2 in the different grouping by age or parasitemia was determined by Mann Whitney test. Associations between immunological outcomes and gender, median parasitemia and age were determined by multivariate regression analysis. Individuals with median parasitemia of 0 or >1 were considered to have low exposure or high exposure, respectively. One Sample t test and Wilcoxon Signed Rank Test were used to compare a k_d_ value with the mean and median of an individual longitudinal k_d_ values, respectively.

The estimation of variation in antibody affinity in each individual was determined by expressing the k_d_ values at any point in time as a proportion of antibody k_d_ value on day 0. Values that fell below 50% or increased above 200% of the day 0 k_d_ values were considered as significant variation in affinity (values below 50% represent significant increase in affinity while values above 200% show significant decrease in affinity). Changes in k_d_ values that are not below 50% or above 200% of the day 0 k_d_ values were considered insignificant and individuals with such changes were considered to have constant k_d_ values.

## Electronic supplementary material

Supplementary Fig 1
