# Supplementary material for: Factors influencing the induction of high affinity antibodies to Plasmodium falciparum merozoite antigens and how affinity changes over time
Source: Sci Rep. 2018 Jun 13;8:9026. doi: 10.1038/s41598-018-27361-w (PMC5998021; doi:10.1038/s41598-018-27361-w)
Supplement: Supplementary file 1 — Supplementary Fig 1 [file 41598_2018_27361_MOESM1_ESM.pdf]

Factors influencing the induction of high affinity antibodies to *Plasmodium falciparum* merozoite antigens and how affinity changes over time

Muyideen K Tijani[1, 2] Sreenivasulu B. Reddy[2] Christine Langer[3] James G Beeson[3] Mats Wahlgren[2] Roseangela I Nwuba[1] Kristina E M Persson[2, 4*]

Supplementary Information

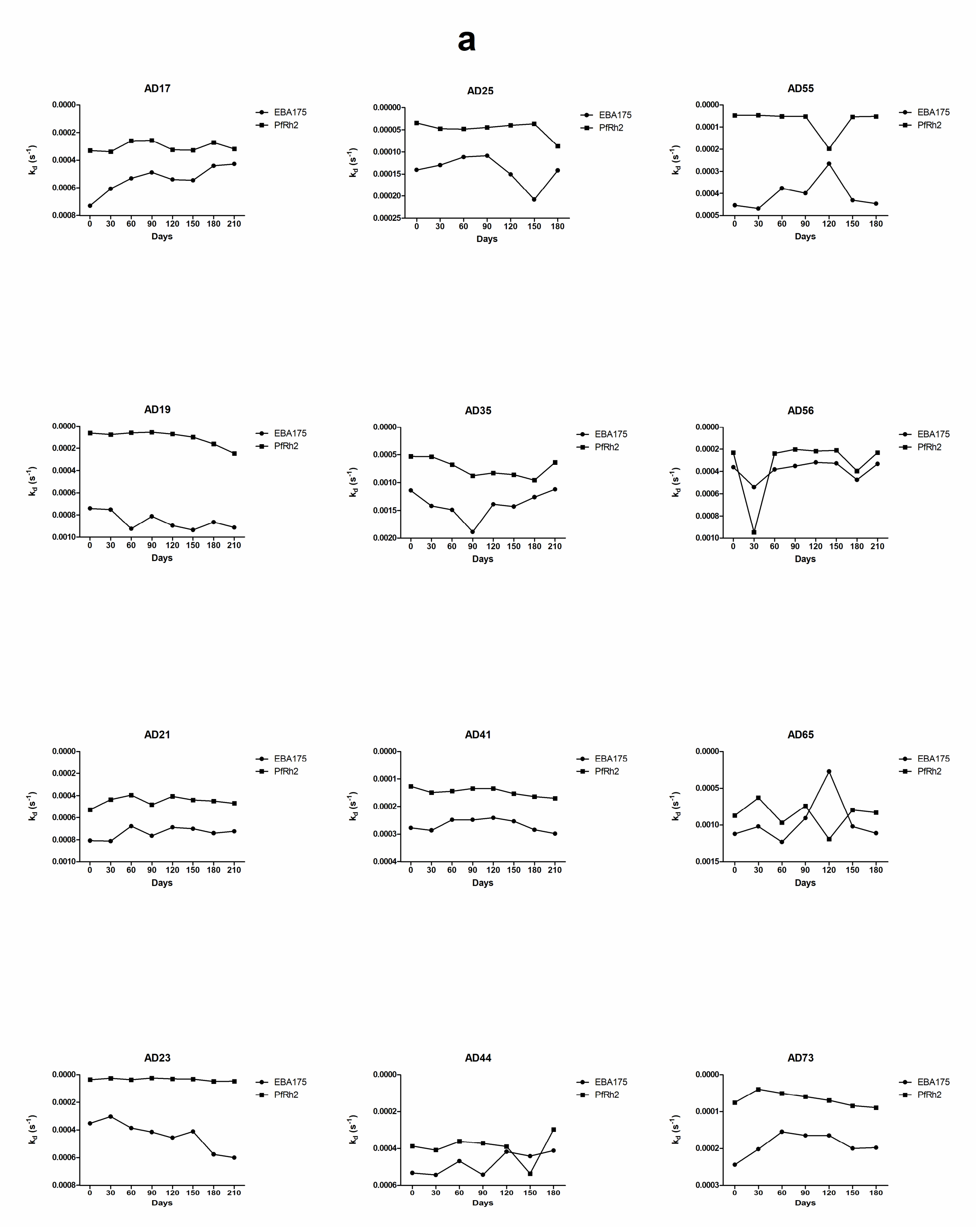

Supplementary Figure 1: EBA175 and PfRh2 antibody affinities (measured as k_d values) of individuals over time.

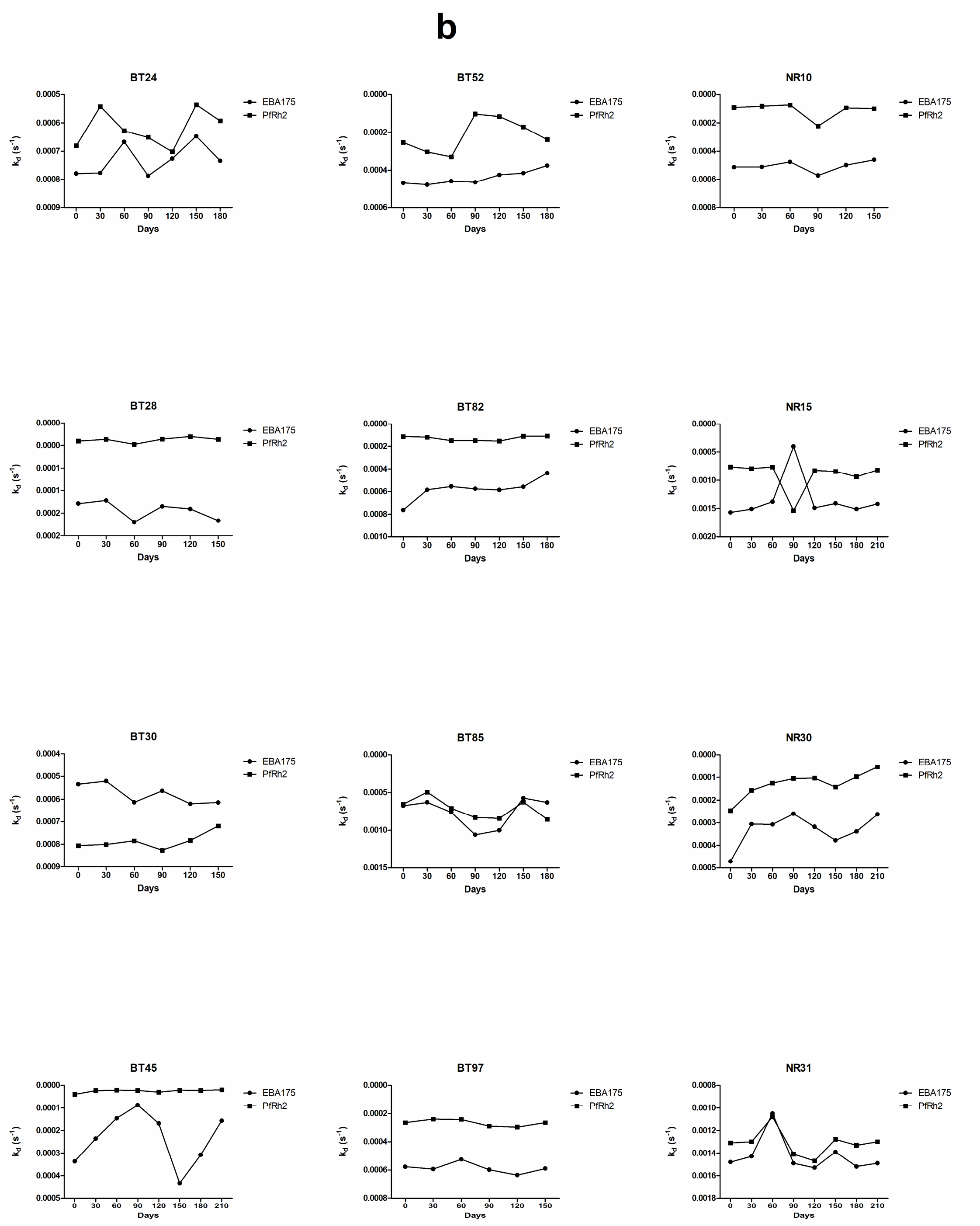

Supplementary Figure 1: EBA175 and PfRh2 antibody affinities (measured as k_d values) of individuals over time.

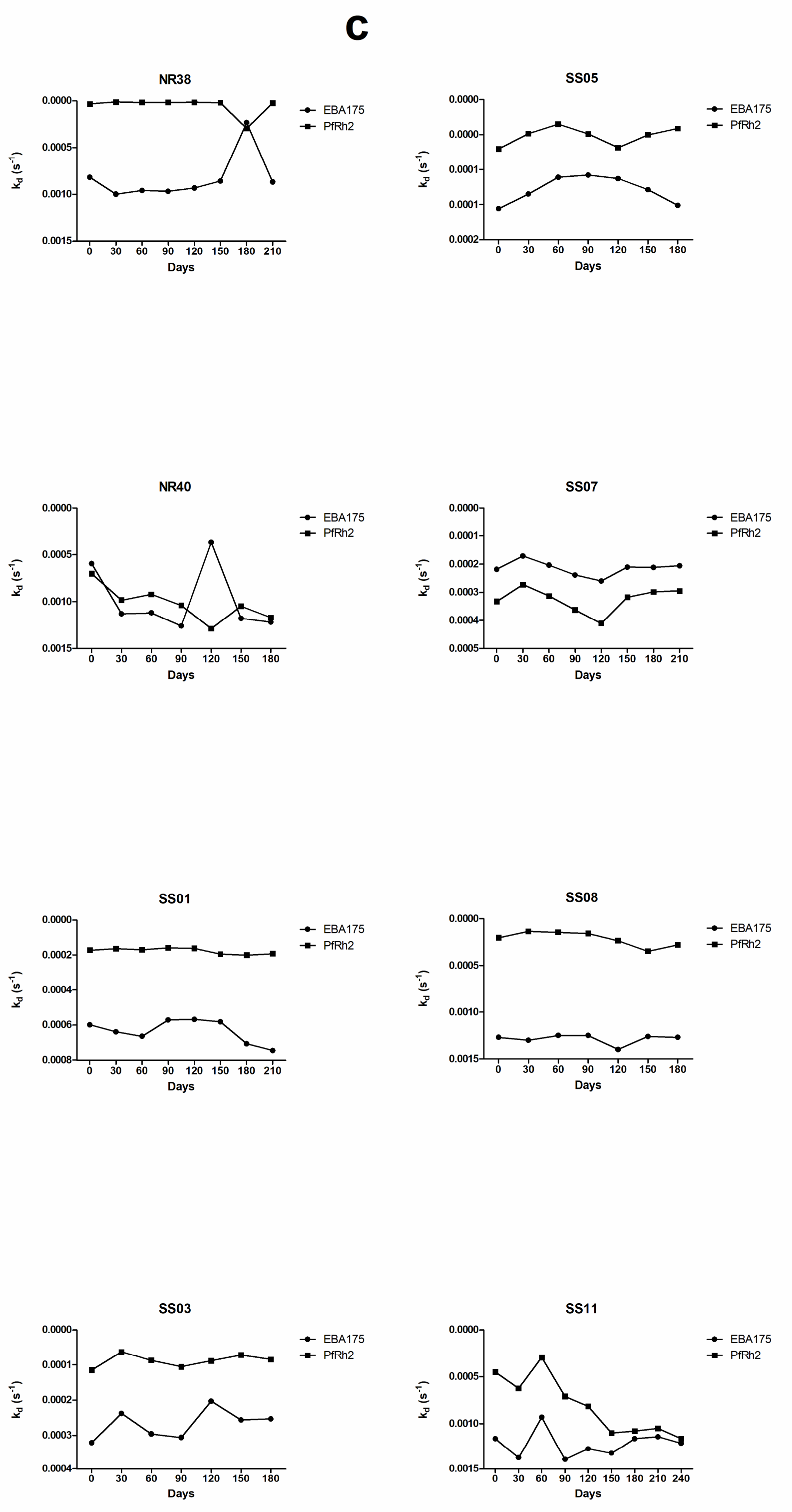

Supplementary Figure 1: EBA175 and PfRh2 antibody affinities (measured as k_d values) of individuals over time.
